# Blood Donor Deferral Patterns at a Tertiary Care Hospital in Central India: A Retrospective Cross-Sectional Study

**DOI:** 10.7759/cureus.116526

**Published:** 2026-09-19

**Authors:** Aditi Dad, Sunil Kumar Patidar, Komal Singh Likhar, Reetesh Kumar Gujar

**Affiliations:** 1 Department of Pathology, Dr. Laxminarayan Pandey Government Medical College, Ratlam, IND

**Keywords:** blood donation analysis, blood donor, donor deferral, hemoglobin, temporary deferral, transfusion medicine

## Abstract

Background and aim

Blood donor deferral is an essential safeguard for donors and recipients, but it also reduces the immediately available donor pool. Because deferral patterns reflect local demographics, health conditions, and screening practices, center-level data are important for donor recruitment and retention planning. The aim of this study was to evaluate the frequency, pattern, and causes of blood donor deferral among prospective donation presentations at a tertiary care teaching hospital in Central India.

Methods

A retrospective, record-based cross-sectional study was conducted at the Blood Centre of Dr. Laxminarayan Pandey Government Medical College, Ratlam. A recorded donation presentation was defined as an age-eligible prospective donor entered after initial age screening into either the Main Register following successful donation or the Deferral Register following pre-donation deferral. Individuals outside the center’s routine age criterion of 18-60 years were screened at the initial counter and were not entered into either register. All 11,354 recorded donation presentations between March 2024 and March 2026 were included. Hemoglobin was screened using the copper sulfate specific-gravity method, with a minimum eligibility threshold of 12.5 g/dL for both male and female donors; the same screening procedure was used in in-house and blood donation camp sessions. Deferral reasons were standardized into predefined categories and classified as temporary or permanent; where more than one reason was documented, all recorded reasons were retained in the reason-specific analysis. Associations of sex, age group, and study period with deferral were assessed using Pearson’s chi-square test or Fisher’s exact test, as appropriate.

Results

Of 11,354 recorded donation presentations, 216 (1.9%; 95% CI, 1.7-2.2%) resulted in deferral. There were 10,219 male presentations (90.0%) and 1,135 female presentations (10.0%). Deferral rates did not differ significantly between male (188/10,219, 1.8%) and female (28/1,135, 2.5%) presentations (p = 0.142). Most deferrals were temporary (193/216, 89.4%), while 23/216 (10.6%) were permanent. Self-reported alcohol intake within the preceding 72 hours (37/193, 19.2%) and low hemoglobin (27/193, 14.0%) were the leading temporary causes, while cardiovascular disease was the most frequent permanent cause (7/23). Deferral rates were 73/3,143 (2.3%) in March-December 2024, 70/6,233 (1.1%) in January-December 2025, and 73/1,978 (3.7%) in January-March 2026 (χ²(2) = 57.15, p < 0.001).

Conclusions

Among recorded donor presentations after initial age screening, 216/11,354 (1.9%) resulted in deferral, and most deferrals were temporary. Recent alcohol intake and low hemoglobin were the most frequent temporary causes and represent practical targets for pre-donation guidance, appropriate clinical evaluation when indicated, and structured reentry of temporarily deferred donors.

## Introduction

Safe blood supply depends on selecting donors who can donate without undue risk to themselves or to recipients. Donor history assessment, physical examination, hemoglobin testing, and application of standardized eligibility criteria remain central to blood safety even when sensitive testing for transfusion-transmissible infections is available [1,2].

Deferral is therefore an essential safety intervention, but it carries operational consequences. Temporary or permanent exclusion reduces the immediately available donor pool, and the experience of being deferred may affect a donor’s subsequent willingness to return. Deferral patterns are also highly center-specific because they reflect local demographics, disease epidemiology, donor recruitment practices, and the way eligibility criteria are implemented [3,4].

Indian studies demonstrate substantial heterogeneity. Reported deferral rates include 3.77% at a Central Indian tertiary hospital, 5.85% at a Chennai tertiary hospital, approximately 7% at a recently established rural blood center, and 16% at a North Indian tertiary institute [3,5-7]. The dominant causes also vary: low hemoglobin is frequently reported as the leading temporary cause, whereas alcohol-related deferral has been prominent in some Central Indian cohorts [3,5,6]. This variability, also demonstrated in other Indian single-center analyses, supports center-level surveillance rather than assuming that a single national profile applies uniformly [8].

The present study evaluated donation presentation records from a tertiary care teaching hospital in Ratlam, Madhya Pradesh. The primary objective was to estimate the proportion of presentations resulting in donor deferral. Secondary objectives were to describe demographic patterns, standardize and classify the documented reasons for deferral, and assess associations of sex, age group, and study period with deferral status.

## Materials and methods

Study design and setting

This retrospective, cross-sectional record review was conducted at the Blood Centre, Department of Pathology, Dr. Laxminarayan Pandey Government Medical College, Ratlam, Madhya Pradesh, India. The study covered donation presentations recorded from March 2024 through March 2026. Complete enumeration was used: all eligible presentation records in the study window were included in the analytic dataset.

Study participants

Inclusion Criteria

For this study, a recorded donation presentation was defined as an age-eligible prospective donor documented in either the Main Register following successful donation or the Deferral Register following pre-donation deferral. Age was screened at the initial counter before entry into either register, using the center’s routine age criterion of 18-60 years. Individuals younger than 18 years or older than 60 years were not entered into either register and therefore could not be enumerated retrospectively. The analytic denominator of 11,354 consequently represents all recorded donor presentations after initial age screening. The same registration and screening workflow was followed for both in-house and blood donation camp sessions.

Exclusion Criteria

Records missing information required for a specific analysis were to be excluded from that analysis. After data cleaning, no registered presentation records were missing the core demographic or outcome variables; therefore, all 11,354 recorded presentations available after initial age screening were retained in the final analysis. Individuals younger than 18 years or older than 60 years had been screened at the initial counter before entry into either source register; consequently, their number and outcomes could not be retrospectively ascertained.

Study size and sampling method

The study included all 11,354 recorded donation presentations available in the Main Register and Deferral Register after initial age screening during the study window. No registered presentation records were excluded for missing core demographic or outcome data. The number of individuals screened out on age grounds before register entry could not be determined because no source register record was created for these individuals. Because complete enumeration was used, no sampling calculation was performed.

Data collection

Data were abstracted from the Blood Centre donor registers into a structured digital master chart covering all 11,354 donation presentations from March 2024 through March 2026. Recorded variables included a serial record identifier, sex, age, donation outcome (accepted donation vs deferral), the documented primary reason for deferral, temporary/permanent classification, and calendar/study period. Documented deferral reasons were standardized into predefined categories and classified as temporary or permanent according to WHO donor selection guidance and the applicable national and institutional donor selection criteria [1,2]. Hemoglobin screening was performed using the copper sulfate specific-gravity method, with a minimum eligibility threshold of 12.5 g/dL for both male and female donors. The same hemoglobin screening method and eligibility threshold were used for both in-house and blood donation camp sessions. Blood pressure and hypertension history were assessed during donor screening. Donors were asked about a history of hypertension, current antihypertensive medication, recent initiation or change in antihypertensive therapy, and associated cardiovascular disease, including previous myocardial infarction. A screening blood pressure of ≥140/90 mmHg was considered elevated. Hypertension alone was not considered an automatic permanent deferral criterion. Donors with adequately controlled blood pressure while receiving stable antihypertensive therapy could be accepted if they fulfilled the remaining donor selection criteria. Donors who had recently initiated antihypertensive therapy or undergone a recent change in antihypertensive dosage were temporarily deferred until blood pressure was stabilized. Permanent deferral was applied when hypertension was associated with cardiovascular disease or another condition meeting the center’s permanent deferral criteria, rather than on the basis of an isolated elevated blood-pressure reading. Data were organized in Microsoft Excel (Microsoft Corporation, Redmond, WA, USA) and analyzed in Python (Python Software Foundation, Wilmington, DE, USA).

Data cleaning and quality control

As part of routine data quality verification, the digitized sex field was compared with the original blood bank registers. Six records (0.05% of 11,354) were reconciled with the source documentation before final analysis. The source registers were accurate, and the validated final distribution was 10,219 male (90.0%) and 1,135 female (10.0%) presentations. To further assess transcription accuracy, 1,200 of the 11,354 analytic records (10.57%) were selected using random numbers and rechecked against the corresponding source registers. No discrepancies were identified for age (0/1,200), sex (0/1,200 after the preceding sex-field correction), or donation outcome (0/1,200). In addition, all 216 deferred records were rechecked against the original Deferral Register for the documented deferral reason and temporary/permanent classification. One deferral-reason transcription discrepancy was identified (1/216, 0.46%), whereas no discrepancies were identified in temporary/permanent classification (0/216). All discrepancies identified during source verification were corrected in the research dataset. For analysis of reasons for deferral, all reasons documented in the Deferral Register were considered. The register contained a free-text reason field rather than a formal multireason coding system. Five of the 216 deferred records contained more than one documented reason; all documented reasons in these records were retained in the reason-specific analysis.

Primary variables

The main study variables were age, sex, deferral status (accepted donation vs deferred), deferral type (temporary vs permanent), and the primary documented reason for deferral. For subgroup analyses, presentations were categorized by sex, age group (18-30, 31-45, and 46-60 years), and study period.

Statistical methods

Categorical variables were summarized as frequencies and percentages. Categorical variables were summarized as frequencies and percentages. The 95% CI for the overall deferral proportion was calculated using the Wilson score method. The donation presentation was the unit of analysis. The donation presentation was the unit of analysis. Unique donor identifiers were not available consistently across all presentation records; therefore, repeated presentations by the same individual could not be identified, and the inferential analyses treated presentations as independent observations. Pearson’s chi-square test was used to assess associations of sex, age group, and study period with deferral status. Fisher’s exact test was used to assess the association between sex and deferral type because an expected cell count was below five. For the two-sided Fisher’s exact test, the scipy.stats.fisher_exact convention was used, in which the p-value is calculated by summing the probabilities of tables with null hypothesis probabilities less than or equal to that of the observed table. All tests were two-sided, with statistical significance set at p < 0.05. Analyses were performed using Python 3.13, pandas version 3.0.1, and SciPy version 1.17.1.

Ethical considerations

The Institutional Ethics Committee of Dr. Laxminarayan Pandey Government Medical College, Ratlam approved this retrospective study (GMC RATLAM/2026/IEC/Approval/513; committee review dated July 16, 2026; approval dated July 28, 2026).

## Results

Study cohort and overall deferral

A total of 11,354 donation presentations from March 2024 through March 2026 were analyzed. Of these, 11,138 (98.10%) resulted in successful donation, and 216 (1.90%; 95% CI, 1.67-2.17%) resulted in deferral. There were 10,219 male presentations (90.0%) and 1,135 female presentations (10.0%). Table 1 summarizes the sex distribution and sex-specific deferral rates.

**Table 1:** Sex distribution and sex-specific deferral rates. Pearson’s chi-square test was used to compare deferral status by sex; χ²(1) = 2.15, p = 0.142. Sex reflects the validated field after source register verification; see Data cleaning and quality control.

Sex	Donation presentations, n	Deferred, n	Deferral rate, %
Male	10,219	188	1.8
Female	1,135	28	2.5
Total	11,354	216	1.9

Deferral rates did not differ significantly between male and female presentations (1.84% vs 2.47%; χ²(1) = 2.15, p = 0.142). The OR for deferral in female vs male presentations was 1.35 (95% CI, 0.90-2.02). Male presentations accounted for 188/216 (87.04%) of all deferrals and female presentations for 28/216 (12.96%), largely reflecting the sex distribution of all presentations.

Age distribution of deferred presentations

Among the 216 deferred presentations, 115 (53.2%) were in the 18-30-year age group, 86 (39.8%) were in the 31-45-year group, and 15 (6.9%) were in the 46-60-year group (Table 2).

**Table 2:** Age distribution among deferred presentations.

Age group	Deferred presentations, n	% of deferred presentations
18-30 years	115	53.2
31-45 years	86	39.8
46-60 years	15	6.9
Total	216	100

When expressed as a proportion of all presentations within each age band, deferral rates were 1.89% at 18-30 years, 1.91% at 31-45 years, and 1.94% at 46-60 years. There was no evidence of an association between age group and deferral status (χ²(2) = 0.009, p = 0.996), indicating that the larger number of deferrals among younger individuals reflected the age distribution of the presentation pool rather than a higher age-specific deferral rate.

Temporary and permanent deferrals

Of the 216 deferred presentations, 193 (89.4%) were classified as temporary and 23 (10.6%) as permanent (Table 3). Among male deferrals, 166/188 (88.3%) were temporary, and 22/188 (11.7%) were permanent; among female deferrals, 27/28 (96.4%) were temporary, and 1/28 (3.6%) was permanent.

**Table 3:** Frequency of temporary and permanent deferrals. Percentages in the male and female columns use the total number of deferrals within each sex as the denominator. Because one expected cell count was below five, Fisher’s exact test was used to assess the association between sex and deferral type; no statistically significant association was detected (two-sided p = 0.324).

Deferral type	All deferrals, n	Male deferrals, n (%)	Female deferrals, n (%)	% of all deferrals
Temporary	193	166 (88.3)	27 (96.4)	89.4
Permanent	23	22 (11.7)	1 (3.6)	10.6
Total deferral	216	188	28	100

Reasons for temporary deferral

Among the 193 temporary deferrals, recent self-reported alcohol intake was the most frequent documented reason (37, 19.2% of temporary deferrals; 17.1% of all deferrals), followed by low hemoglobin (27, 14.0%), antibiotic medication (17, 8.8%), and rabies post-exposure vaccination (15, 7.8%). The complete distribution of all 26 standardized temporary deferral categories is shown in Table 4. Five deferred presentations had more than one documented reason; all documented reasons were retained in the reason-specific analysis, and details of these presentations are provided in Appendix A.

**Table 4:** Reasons for temporary deferral. Temporary/permanent classification was based on WHO donor selection guidance and applicable national and institutional donor selection criteria [1,2]. Because all documented reasons were retained for presentations with more than one reason, reason-specific counts are not mutually exclusive and may sum to more than the number of temporarily deferred presentations; percentages use 193 temporarily deferred presentations as the denominator.

Documented reason for deferral	Deferrals, n	% of temporary deferrals	% of total deferral
Alcohol intake within the preceding 72 hours (self-reported)	37	19.2	17.1
Low hemoglobin	27	14	12.5
Antibiotic medication	17	8.8	7.9
Rabies vaccination	15	7.8	6.9
Allergic reaction/disease	12	6.2	5.6
Recent tattooing	10	5.2	4.6
Skin/fungal infection	11	5.7	5.1
Recent ear piercing	8	4.1	3.7
Low body weight (<50 kg)	7	3.6	3.2
Recent dental extraction	6	3.1	2.8
Tetanus vaccination	5	2.6	2.3
Recent minor surgery	5	2.6	2.3
Recent major surgery	5	2.6	2.3
Steroid medication	4	2.1	1.9
Recent typhoid	4	2.1	1.9
Hypertension	3	1.6	1.4
Recent blood donation (within three months)	3	1.6	1.4
Recent jaundice	3	1.6	1.4
Thyroid medication	2	1	0.9
Recent blood transfusion received	2	1	0.9
Recent malaria	2	1	0.9
Hypoglycemic episode	1	0.5	0.5
Low blood pressure	1	0.5	0.5
Diarrhea	1	0.5	0.5
Abnormal bleeding tendency	1	0.5	0.5
Abnormal uterine bleeding	1	0.5	0.5

Reasons for permanent deferral

Among the 23 permanent deferrals, cardiovascular disease was the most frequent documented cause (7, 30.4%), followed by insulin-dependent diabetes mellitus (4, 17.4%), with kidney disease and hyperthyroidism accounting for three cases each (13.0% each). Screening-detected elevated blood pressure was classified as a temporary deferral when it did not meet criteria for a permanent associated condition. Hypertension alone was not considered an automatic permanent deferral reason at the center. The complete distribution of the nine permanent deferral categories is shown in Table 5.

**Table 5:** Reasons for permanent deferral. Temporary/permanent classification was based on WHO donor selection guidance and applicable national and institutional donor selection criteria [1,2].

Documented reason for deferral	Deferrals, n	% of permanent deferrals	% of total deferral
Cardiovascular disease	7	30.4	3.2
Diabetes mellitus (insulin-dependent)	4	17.4	1.9
Kidney disease	3	13	1.4
Hyperthyroidism	3	13	1.4
Hepatitis B	2	8.7	0.9
History of injecting drug use	1	4.4	0.5
Chronic active vitiligo autoimmune skin disease	1	4.4	0.5
Leprosy	1	4.4	0.5
Chronic lung disease	1	4.4	0.5
Total	23	100	10.8

Variation across study periods

Period-specific deferral rates were calculated directly from the number of presentations in each calendar-based study period. Rates differed across the three periods (χ²(2) = 57.15, p < 0.001): 2.3% during March-December 2024, 1.1% during January-December 2025, and 3.7% during January-March 2026 (Table 6).

**Table 6:** Deferral rates across study periods. Global comparison across the three periods: χ²(2) = 57.15, p < 0.001.

Study period	Presentations, n	Deferred, n	Deferral rate, %
March-December 2024	3,143	73	2.3
January-December 2025	6,233	70	1.1
January-March 2026	1,978	73	3.7
Total	11,354	216	1.9

The three periods differed in duration, and information on camp mix, first-time vs repeat status, seasonal effects, and recruitment intensity was not available for all presentations. The observed variation should therefore be interpreted as a descriptive signal for continued monitoring rather than evidence of a causal temporal trend.

## Discussion

Principal findings

This complete-enumeration analysis of 11,354 donation presentations found an overall deferral rate of 1.90%, with 89.35% of deferrals classified as temporary. Recent alcohol intake and low hemoglobin were the leading temporary causes, cardiovascular disease was the leading permanent cause, and deferral status was not significantly associated with sex or age group. Deferral rates differed across the three study periods.

Comparison with previous studies

The observed deferral rate of 1.9% was lower than several published Indian estimates: Kujur et al. reported 3.77% at a tertiary hospital in Central India, Soundharya et al. reported 5.85% at a tertiary hospital in Chennai, Minj et al. reported approximately 7% at a newly established rural blood center, and Malhotra and Negi reported 16% at a North Indian tertiary institute [3,5-7]. Differences in recruitment setting, donor composition, eligibility criteria, local health patterns, and classification practices likely contribute to this heterogeneity, as also demonstrated in other Indian single-center analyses [8]. The present findings should therefore be interpreted as a center-specific epidemiologic profile rather than a benchmark for other settings.

Temporary deferrals accounted for 89.4% of all deferrals, close to the 88% temporary proportion reported by Malhotra and Negi and consistent with the predominance of temporary deferral described in other Indian series [3,9,10]. This is operationally important because many temporarily deferred individuals may become eligible to donate after the reason for deferral has resolved.

Reason profile and comparison across centers

Self-reported recent alcohol intake was the most frequent temporary deferral reason in this study (19.2%), followed by low hemoglobin (14.0%). This alcohol-led pattern resembles the Central Indian experience reported by Kujur et al., whereas low hemoglobin dominated the Chennai cohort reported by Soundharya et al. [5,6]. The 27 low-hemoglobin deferrals in the present study represent recorded deferrals under the center’s screening protocol and should not be interpreted as an estimate of anemia prevalence in the underlying population. Hemoglobin was screened by the copper sulfate specific-gravity method using a 12.5 g/dL eligibility threshold for both sexes, consistently across in-house and camp sessions. Differences in hemoglobin-screening procedures, eligibility thresholds, donor selection, and register-entry practices across centers may therefore limit direct comparison of low-hemoglobin proportions. Table 7 compares selected standardized temporary deferral categories across these studies.

**Table 7:** Comparison of temporary deferral causes across studies (%). Percentages use each study’s total number of temporary deferrals as the denominator. The total temporary deferrals were 193 in the present study, 764 in Soundharya et al. [6], and 614 in Kujur et al. [5]. Categories not reported in a comparator study are omitted.

Cause for temporary deferral	Present study (n = 193), %	Kujur et al. [5] (Central India), n = 614, %	Soundharya et al. [6] (Chennai), n = 764, %
Low hemoglobin	14	16.7	73.7
Alcohol intake	19.2	32	11.9
Low body weight	3.6	5.5	6
Tattoo within one year	5.2	1.1	2.7
Antibiotic medication	8.8	7.3	1.3
Recent donation within three months	1.6	6.7	0.9
Vaccination (rabies/tetanus)	10.4	1.3	0.7

Vaccination-related deferral was also notable: rabies post-exposure vaccination accounted for 15 cases (7.8% of temporary deferrals) and tetanus vaccination for five (2.6%), together representing 10.36% of temporary deferrals. This proportion was higher than the vaccination-related proportions reported in the two comparator studies [5,6]. The present dataset did not capture the indication for vaccination or local animal-bite incidence, so the reason for this difference cannot be determined from these data.

Among the 23 permanent deferrals in the present study, cardiovascular disease was the most frequently documented reason (n = 7), followed by insulin-dependent diabetes mellitus (n = 4). Other permanent deferral reasons included kidney disease, hyperthyroidism, hepatitis B, a history of injecting drug use, chronic active vitiligo/autoimmune skin disease, leprosy, and chronic lung disease (Table 5). Hypertension alone was not considered an automatic permanent deferral criterion at this center. An isolated screening blood pressure of ≥140/90 mmHg was managed as a temporary deferral in the absence of an associated permanent deferral condition, whereas permanent deferral was applied when hypertension was associated with cardiovascular disease or another condition meeting permanent deferral criteria. Because temporary-vs-permanent classification of hypertension may vary according to the applicable donor selection criteria and local protocol [2,11], permanent deferral proportions for hypertension were not directly compared across centers.

Sex and age patterns

Female presentations accounted for 10.0% of the study population. Deferral rates were 1.84% among male presentations and 2.47% among female presentations, with no statistically significant difference (p = 0.142; female vs male OR 1.35, 95% CI 0.90-2.02). This contrasts with some Indian cohorts in which female donors had higher deferral rates, often related to low hemoglobin [3,5,6].

Source register verification identified six records that had been incorrectly transcribed as male rather than female in the research master dataset; the original blood bank registers were correct, and all six were corrected before the final analysis. A subsequent random audit of 1,200 records found no additional discrepancies in age, sex, or donation outcome, while verification of all 216 deferred records identified one deferral-reason transcription discrepancy and no temporary/permanent classification discrepancies. All identified discrepancies were corrected in the research dataset.

Age group was also not associated with deferral status: rates were approximately 1.9% in each prespecified age band (18-30, 31-45, and 46-60 years; p = 0.996). The greater absolute number of deferrals among younger individuals therefore reflected the larger number of presentations in that age group rather than a higher age-specific deferral rate.

Reason profile and implications for donor retention

Temporary deferral may affect subsequent donor return. Hillgrove et al. reported reduced return after low-hemoglobin deferral, while Custer et al. described broader adverse effects of temporary deferral on future whole-blood donation [12,13]. Subsequent evidence has also shown that donor return varies according to the reason and duration of temporary deferral [14]. Low hemoglobin may also identify a clinically relevant condition that warrants appropriate evaluation rather than being viewed only as an operational barrier to donation [15,16]. A structured pathway that explains the reason and expected duration of deferral, provides appropriate health guidance, and gives a clear reentry date may help retain eligible donors. This is particularly relevant to recent alcohol intake and low hemoglobin, the two leading temporary causes in the present study.

Variation across study periods

Deferral rates differed across study periods, ranging from 1.12% in 2025 to 3.69% in January-March 2026 (χ²(2) = 57.15, p < 0.001). These rates were calculated from the observed number of presentations in each period. However, the periods differed in duration, and the analytic dataset did not permit adjustment for camp mix, seasonality, recruitment intensity, or first-time/repeat donor status. In addition, repeated presentations by the same donor could not be identified, so the period comparison treated presentations as independent. Within-donor correlation may result in underestimated standard errors and overly small p-values; therefore, the statistical significance of the period comparison should be interpreted cautiously. The observed period-specific proportions remain descriptive and should not be interpreted as evidence of a causal temporal trend.

Strengths and limitations

Strengths of this study include complete enumeration of eligible donation presentations in the study window, source register verification of the sex field, a random audit of 1,200 analytic records, and verification of all 216 deferred records, transparent reporting of all standardized deferral categories rather than use of a residual “other” category, and presentation-level denominators for the study-period analysis.

Several limitations should be considered. First, this was a retrospective, single-center study, so the findings may not generalize to other donor populations or screening practices. In addition, individuals outside the center’s routine age criterion were screened at the initial counter before entry into either the Main Register or the Deferral Register, and their number could not be reconstructed from the retrospective records. Accordingly, the observed 1.90% deferral proportion applies to recorded donor presentations after initial age screening and may underestimate the proportion of all individuals initially approaching for donation who were found ineligible. Second, the unit of analysis was the donation presentation rather than the unique donor. Unique donor identifiers were not consistently available across all presentation records, so repeated presentations by the same individual could not be linked, and the inferential analyses treated presentations as independent. Correlation among repeated presentations may lead to underestimated standard errors and anti-conservative p-values. This limitation is principally relevant to the statistically significant comparison across study periods; the nonsignificant associations of sex and age group with deferral status are not rendered statistically significant by accounting for such clustering and should continue to be interpreted as null findings. Third, the center routinely used an age criterion of 18-60 years during the study period. Individuals outside this age range were screened at the initial counter before entry into either the Main Register or the Deferral Register, and their number and outcomes could not be retrospectively ascertained. Because applicable national guidance permits some eligible repeat whole-blood donors beyond 60 years and up to 65 years, the present findings do not characterize potentially eligible donors aged 61-65 years; the findings therefore do not characterize eligible donors aged 61-65 years. Fourth, exact hemoglobin values, blood pressure measurements, and other vital signs in the paper registers were not transcribed into the analytic dataset, limiting more granular assessment of the leading deferral conditions. Fifth, five records with concurrent screening findings were assigned to one predominant reason, which may understate secondary contributors. Finally, differences in camp mix, seasonality, recruitment intensity, and donor history across study periods could not be controlled, and the observational design does not support causal inference.

## Conclusions

Blood donor deferral at this tertiary care center in Central India was infrequent overall and predominantly temporary. Recent alcohol intake and low hemoglobin were the most frequent temporary deferral reasons, while cardiovascular disease was the leading permanent deferral reason.

The most actionable opportunities are to reduce preventable presentation-related deferrals where feasible and to support the safe return of temporarily deferred donors. Clear pre-donation guidance regarding recent alcohol intake, appropriate evaluation and management of low hemoglobin, explicit reentry instructions, and continued monitoring of period-specific deferral rates may strengthen donor retention and blood-supply resilience at this center.

## Appendices

Appendix A 

**Table 8:** Deferred presentations with more than one documented reason for deferral. The Deferral Register contained a free-text reason field rather than a formal multi-reason coding system. Five of the 216 deferred presentations contained more than one documented reason. All documented reasons in these five records were retained in the reason-specific analysis. Research dataset serial numbers are shown only for traceability within the de-identified study dataset.

Supplementary record	Research dataset serial no.	Documented reasons
1	160	Low body weight (<50 kg), fever, low hemoglobin, allergic reaction
2	161	Low body weight (<50 kg), low hemoglobin
3	162	Low body weight (<50 kg), low hemoglobin
4	166	Major surgery, blood transfusion
5	177	Underweight/low body weight and low hemoglobin
